# The Potential of Using Shungite Mineral from Eastern Kazakhstan in Formulations for Rubber Technical Products

**DOI:** 10.3390/ma18010114

**Published:** 2024-12-30

**Authors:** Sergey V. Nechipurenko, Valeriya V. Bobrova, Andrey V. Kasperovich, Mubarak Ye. Yermaganbetov, Sergey A. Yefremov, Aigerim K. Kaiaidarova, Danelya N. Makhayeva, Bayana B. Yermukhambetova, Grigoriy A. Mun, Galiya S. Irmukhametova

**Affiliations:** 1 Faculty of Chemistry and Chemical Technology, Al-Farabi Kazakh National University, Almaty 050040, Kazakhstan; nechipurenkos@mail.ru (S.V.N.); efremsa@mail.ru (S.A.Y.); aigerim_ko@list.ru (A.K.K.); danelya.1993@gmail.com (D.N.M.); baya_yerm@mail.ru (B.B.Y.); mungrig@yandex.ru (G.A.M.); 2 National Engineering Academy of the Republic of Kazakhstan, Almaty 050010, Kazakhstan; 3 Department of Polymer Composite Materials, Faculty of Technology of Organic Substances, Belarusian State Technological University, 220006 Minsk, Belarus; bobrova@belstu.by (V.V.B.); andkasp@belstu.by (A.V.K.); 4 K. Turysov Institute of Geology and Oil and Gas Engineering, Satbayev University, Almaty 050000, Kazakhstan; m.yermaganbetov@satbayev.university

**Keywords:** shungite mineral, industrial rubbers, Mooney viscosity, kinetic parameters, elastic-strength properties, relative residual compression set, aggressive media

## Abstract

This study examined the effect of partially replacing semi-reinforcing carbon black grade N550 (up to 10 pts. wt.) and fully replacing industrial chalk with natural shungite mineral in industrial formulations of elastomer compositions intended for manufacturing various rubber technical products. It has been shown that due to the high content of carbon and silicon components in the composition of shungite mineral micropowders, their use as a filler in elastomer formulations significantly improves the physical and mechanical properties of rubber technical products (RTPs) manufactured using such compositions. It was determined that the use of SM as a partial replacement for carbon black in rubbers intended for molded rubber technical products contributes to a reduction in Mooney viscosity (up to 26.8%) and optimal vulcanization time (up to 23.7%), achieving rubbers with the required set of physical–mechanical properties and with an enhancing sealing capability (up to 19.7%). It has been established that the use of shungite mineral micropowders as a complete replacement for industrial chalk increases the strength of rubber products (RTPs) by up to 18.5% and enhances their resistance to liquid aggressive environments.

## 1. Introduction

Carbon black and white silica (hydrated silicon dioxide, mSiO_2_•nH_2_O) have traditionally been the most common fillers (reinforcing additives) for elastomer composites based on natural and synthetic rubbers [1]. The incorporation of these fillers into elastomers significantly improves their mechanical properties, particularly their strength and deformation characteristics [2,3]. The reinforcement of elastomers by these fillers is attributed to their high specific surface area, nanoscale size, and the presence of active functional groups on their surface. Due to its strong interactions with hydrocarbon rubbers, carbon black exhibits strong reinforcing effects and has been used as a reinforcing filler since 1904. However, the use of carbon black has become a pressing issue due to its petroleum-based origin and various adverse effects on human health and the environment [4]. Compared to carbon black, silica particles are less compatible with unsaturated hydrocarbon rubbers and tend to form a “filler–filler” network due to hydrogen bonding between silanol groups present on the surface of the particles [5,6].

In response to the needs of the rubber industry and the necessary protection of the environment, continuous research is being conducted to identify other types of fillers of different origins but with characteristics similar to or superior to those of carbon black. One promising direction is the use of natural mineral materials as well as plant-based raw materials [7,8,9,10,11,12,13,14,15,16,17,18,19,20,21,22,23,24,25,26]. The introduction of mineral fillers into rubber not only enhances its physical–mechanical properties, but also imparts several additional important operational qualities, including increased thermal stability, fire resistance, low diffusion permeability, environmental friendliness, and relatively low production cost [27,28,29,30,31,32,33]. Essentially, these materials are complex, structurally heterogeneous systems consisting of a low-modulus, highly elastic matrix into which much stiffer and stronger particles of dispersed filler are embedded. Such materials exhibit complex mechanical behaviors (finite deformations, nonlinear elasticity, and viscoelasticity) due to the different natures of the reversible and irreversible structural changes that occur during deformation. Currently, polymer and elastomer composites with various mineral fillers are the subject of intensive research, both experimental and theoretical [34,35,36,37,38,39]. In terms of application, the most promising use of these materials is in the production of automotive tires and rubber technical products.

## 2. Materials and Methods

### 2.1. Materials

**Figure 1:**
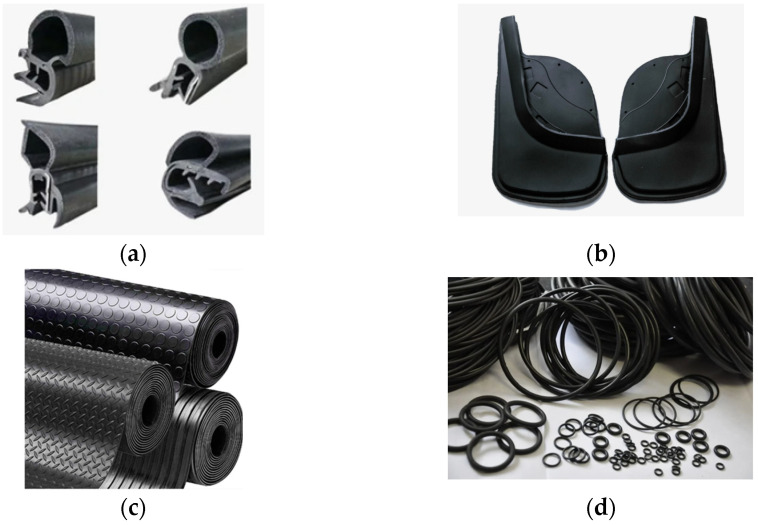
Photo of the RTPs: (**a**) profiles; (**b**) mudguards; (**c**) mats; (**d**) sealing rings.

For the production of industrial elastomer compositions used in the manufacturing of molded rubber technical products (RTPs), the following rubbers were utilized (Figure 1): butadiene-styrene rubber produced via emulsion polymerization (SBR), ethylene-propylene rubber (SKEP), isoprene rubber (IR), and special-purpose butadiene-nitrile rubber BNR-12AN (the rubbers were produced in the Russian Federation JSC “Krasnoyarsk Synthetic Rubber Plant”). The formulations of the studied rubbers are presented in Table 1.

In addition to rubber, which is the main component of industrial rubber compounds, other ingredients are present. Among them are carbon black and chalk. Carbon black grade N550 (Table 2) is characterized by medium particle dispersion and acts as a semi-reinforcing filler. It provides a high extrusion capability, relatively high tear resistance, high modulus and hardness, and low swelling.

Most mineral fillers are low structure and have an undeveloped surface; they do not form chain structures or contribute to gel formation in compositions. Fine hydrophobic chalk mixes well with rubbers, provides improved processing properties to the mixtures, and has virtually no effect on the scorching and vulcanization of rubber compounds. Additionally, the use of chalk enhances resistance to external influences, UV radiation, and chemical solvents and prevents cracking, thereby improving elasticity. An important function of fine hydrophobic chalk as a filler in rubber products is the reduction in production costs. Due to its low cost, it significantly reduces overall expenses. Besides cost reduction, fine hydrophobic chalk enhances the quality of rubber, extending its shelf life and preventing aging. In raw rubbers, fine chalk is also used as a dusting agent. It facilitates the forming process of rubber products.

Chalk mainly consists of CaCO_3_ (97–99%) with impurities (sand, iron, and aluminum oxides). The characteristics of the chalk used in the studied industrial rubber compounds are shown in Table 3.

The requirements for the molded RTPs were set according to the technical specifications for each product type. The requirements for the studied rubbers are presented in Table 4.

Molded rubber technical products (RTPs) must meet the requirements specified in the technical conditions for each product type. The requirements for the studied rubbers are presented in Table 4. It is worth noting that general-purpose rubber is used for producing rubber profiles, mudguards, and mats, while specialized butadiene-nitrile rubbers, such as BNR-12AN, are used for RTPs like sealing rings.

**Figure 2:**
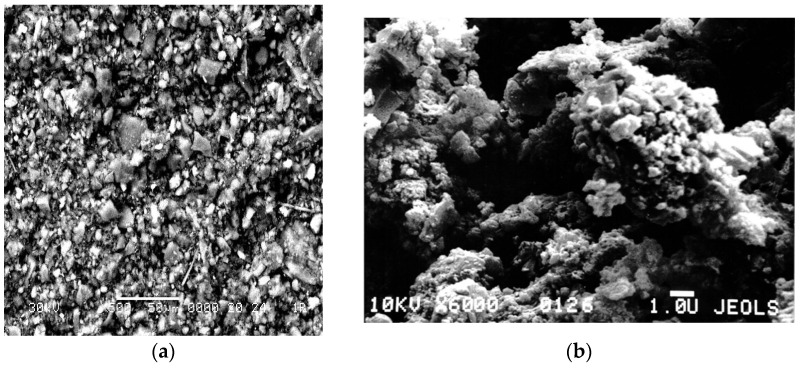
SEM images of SM at different magnifications: (**a**) ×500, (**b**) ×6000.

The shungite mineral (SM) used in this work is a highly dispersed micropowder characterized by a high content of carbonaceous and silicon components. It was produced from ore sourced from East Kazakhstan, Republic of Kazakhstan. It is an unusual natural composite with uniformly distributed, highly dispersed crystalline particles within an amorphous carbon matrix, as shown in Figure 2. There is a strong bond between the carbon and silicate components. This mineral is characterized by a high density and chemical resistance.

Due to the unique composition and structure of the original rock, SM is inherently a carbon–silicon material with bipolar properties and exhibits a range of unusual physical, chemical, and technological characteristics. Even ultrafine particles consist of two phases: a carbon phase and a complex silicate phase. As a result, this mineral can interact with both polar and non-polar substances. SM mixes easily and forms stable homogeneous mixtures with all types of binders, whether organic or inorganic (aqueous suspensions, rubbers, fluoroplastics, resins, cements, etc.). The high compatibility of the mineral with binders enables the creation of highly filled compositions, including rubber-based ones.

The physical–chemical properties and elemental composition of shungite mineral are presented in Table 5 and Table 6, respectively.

The shungite mineral from Eastern Kazakhstan can be classified as a medium-carbon shungite rock, as it contains 17.16% *w/w* carbon and a mineral siliceous base (16.52% *w/w* Si) (average value from 5 samples). Therefore, it was reasonable to compare the effect of SM on the properties of elastomer compositions with that of both carbon black and chalk.

In this work, a partial replacement (5.0 and 10.0 pts. wt., experimental mixture) of the semi-reinforcing carbon black grade N550 with SM was carried out in compositions intended to produce rubber profiles and sealing rings. Additionally, rubber formulations for mudguards, profiles, and mats were developed with a complete replacement of industrial chalk with the studied mineral SM (experimental mixture) to reduce the cost of the finished products and potentially improve the specific properties of the RTPs (Table 7).

### 2.2. Testing

#### 2.2.1. Determination of the Plastoelastic Properties of the Rubber Compounds

During the study, the Mooney viscosity of rubber compounds was determined using an MV 2000 shear disc viscometer (Alpha Technologies, Hudson, OH, USA) in accordance with the standard methodology [41]. The Mooney viscosity of rubber compounds is defined as the degree of resistance to the rotation of a cylindrical metal rotor immersed in a sample placed in a test chamber.

#### 2.2.2. Determination of Vulcanization Kinetics of Rubber Compounds

The kinetic parameters of rubber compound vulcanization were determined by measuring the torque during shear deformation induced by the oscillations of a biconical rotor with a specific frequency and amplitude at the set temperature of the rubber compound sample [42]. The following indicators were derived from the resulting rheological curves to characterize the rheological and vulcanization properties of the compounds:

M_L_—minimum torque, dN·m;

M_H_—maximum torque, dN·m;

t_s2_—time required for the minimum torque to increase by 2 units, min;

t_90_—time to reach optimal vulcanization, min;

ΔM—difference between maximum and minimum torque, dN·m.

Testing was conducted using an ODR 2000 rheometer (Alpha Technologies, Hudson, OH, USA).

The vulcanization process for elastomer compositions based on general-purpose rubbers was performed at 143 °C for 30 min, and for special-purpose rubbers at 153 °C for the same duration [42].

#### 2.2.3. Methodology for Determination of the Physical–Mechanical Indicators of Vulcanizates

The elastic-strength properties of elastomer compositions were evaluated based on tensile strength and elongation at break, determined using a T2020 DC10 SH tensometer (Alpha Technologies, Hudson, OH, USA). The tests were conducted according to the standard procedure [43]. The test result was recorded as the arithmetic mean of the indicators from no fewer than five tested samples from a single batch of the rubber compound.

The Shore A hardness of elastomer compositions was determined using the standard methodology [44] using a DIGI-TEST durometer (Bareiss, Oberdischingen, Germany).

#### 2.2.4. Determination of Resistance of Rubber to Aggressive Factors

The resistance of rubber to thermal aging in an air environment was evaluated by measuring the changes in tensile strength and elongation at break after exposure in a thermostat at 100 ± 2 °C for 72 ± 1 h for general-purpose rubbers and at 125 °C for 72 ± 1 h for special-purpose rubbers. The tests were conducted according to the standard methodology [45].

The resistance of elastomer compositions to liquid aggressive media was assessed by measuring the changes in mass and volume of the samples after immersion in a standard liquid. The chemical resistance of the rubber was studied using a mixture of isooctane and toluene in a 70:30 volume ratio for 1–7 days at a temperature of 23 ± 2 °C, in accordance with the standard methodology [46].

#### 2.2.5. Determination of Relative Residual Compressive Deformation (RRCD)

The determination of the relative residual compressive deformation (RRCD) of the rubber was performed according to [47]. The result of the test was recorded as the arithmetic mean of at least three measurements.

The *RRCD* of rubbers was determined from nine samples and calculated as a percentage with an accuracy of 0.01 using Formula (1):

(1) $$RRCD=\frac{h_{0}-h}{h_{0}-h_{S}}*100\%$$

where

*h*_0_—initial height of the sample before testing, mm;

*h*—height of the sample after “relaxation”, mm;

*h_s_*—height of the stopper, mm.

The essence of the method is that the samples are subjected to static compression deformation for 24 h, and the RCS value is used to determine the ability of the rubber to retain its elastic properties after aging in a compressed state under specific conditions (25% compression at a temperature of 125 ± 2 °C). The test result was recorded as the arithmetic mean of the values for all the samples (at least three).

The structure and morphology of the natural fillers used were analyzed with an S-4800 scanning electron microscope (Hitachi, Tokyo, Japan). Structural analysis of the samples was performed using an EDX Quantox-200 fluorescence spectrometer (Bruker, Hanau, Germany).

## 3. Results and Discussion

### 3.1. Technological Properties

Since rubber compounds must possess the required performance properties and be processable, incorporating new ingredients into elastomer formulations can significantly influence the set of technological properties that determine not only the processing parameters but also the quality of the resulting semifinished products.

#### 3.1.1. Investigation of Mooney Viscosity of Rubber Compounds

**Figure 3:**
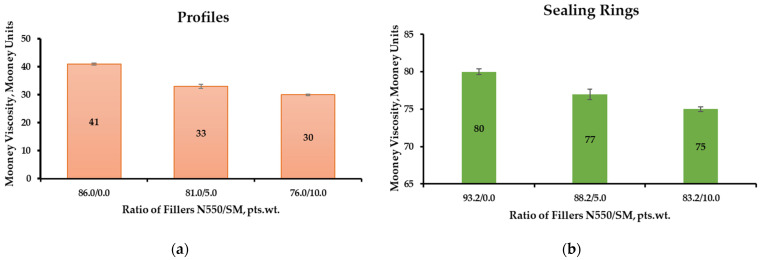
Dependence of Mooney viscosity changes on the dosage of SM in partial replacement of N550 in rubber compounds intended for (**a**) rubber profiles and (**b**) sealing rings.

In formulations based on a combination of the rubbers SKEPT-50 + SBR-30 ARKM-15, intended for rubber profiles, it was found that replacing 5.0 and 10.0 pts. wt. of carbon black grade N550 with SM resulted in a 19.5% and 26.8% decrease in the Mooney viscosity of the rubber compounds (Figure 3a). In the formulations based on the special-purpose rubber NBR-12AN, intended for sealing rings, this indicator decreased slightly, up to 6.3%, when carbon black was replaced (Figure 3b). This behavior may be attributed to the nature of the mineral, specifically the physical–chemical properties of its surface, which influence its interaction with different rubber matrices. 

**Figure 4:**
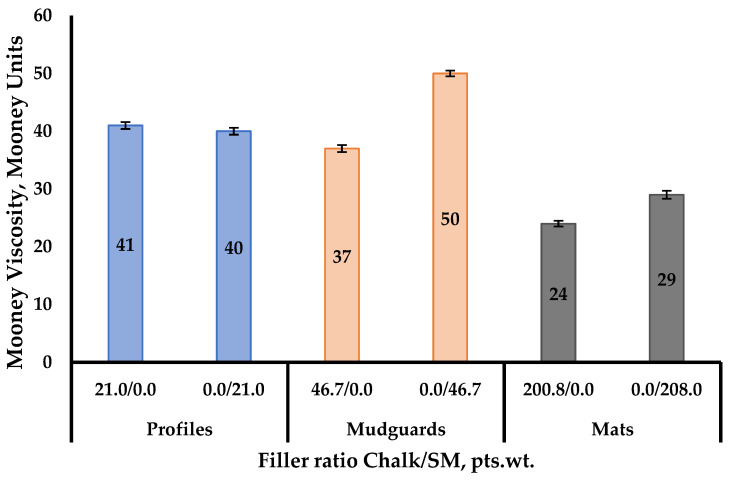
Changes in the Mooney viscosity when chalk was fully replaced with SM in rubber compounds intended for the production of rubber mats, mudguards, and profiles.

In the case of complete replacement of industrial chalk with SM, it was found that the Mooney viscosity of the rubber compounds for profiles remained practically unchanged (the change was 1 Mooney unit). At the same time, the use of SM in the formulations for rubber mudguards and mats increased this parameter by 35.1% and 20.8%, respectively, compared to industrial rubber compounds (Figure 4).

The obtained results indicate that in compositions based on SKI-3 + SBR-30 ARKM-15, the studied mineral interacts more actively with the components of the rubber compounds, which will likely lead to improved performance characteristics of the finished products.

#### 3.1.2. Determination of the Kinetic Parameters of the Vulcanization Process

A comparative analysis of the kinetic parameters of the vulcanization process for rubber compounds that was used in profile production (Table 8) revealed that introducing shungite mineral at dosages up to 10.0 pts. wt. reduced the time to reach the optimal vulcanization level (*t*_90_) by 10.9–23.7%, thereby shortening the vulcanization process for rubber technical products. At the same time, the use of SM decreased the resistance of the rubbers to premature vulcanization (*t_s_*_2_) by 21.2–30.1% and reduced the Δ*M* parameter [48], which indirectly characterizes the crosslinking density of rubbers, by 17.4–26.8% compared to the industrial mixture.

For compositions intended for the production of sealing rings, replacing carbon black with SM resulted in minor changes in the *t*_90_ and t_s2_ parameters and a reduction in crosslinking density by up to 12.9%.

The use of SM as a full replacement for chalk in the formulations of rubber compounds intended for the production of rubber profiles, mudguards, and mats (Table 9) had virtually no effect on the kinetic parameters of vulcanization (changes in the studied characteristics were within 6.1%). This is likely to contribute to maintaining the technological processing parameters of these rubber compounds (Table 9).

### 3.2. Physical–Mechanical and Performance Properties of the Studied Elastomer Compositions

#### 3.2.1. Investigation of Elastic and Strength Properties of Rubbers

Strength is one of the primary characteristics of structural materials, determining the material’s resistance to failure under mechanical loads. The strength of rubbers is significantly influenced by the type and microstructure of the rubber, the type of vulcanizing system, the nature of the structures formed during vulcanization, as well as the concentration and morphological characteristics of the fillers, plasticizers, and modifiers, the testing conditions, and other factors [49].

To evaluate the elastic and strength properties of rubbers based on SKEPT-50 + SBR-30 ARKM-15 (profiles) and BNKS-28AN (sealing rings) containing SM as a partial replacement for N550 carbon black, the indicators of tensile strength (*f*_*p*_) and elongation at break (*ε*_*p*_) were determined (Table 10). The analysis of the primary elastic and strength properties of rubbers before thermal aging showed that introducing SM into rubber compounds as a replacement for semi-reinforcing carbon black N550 in the studied dosages allowed for the production of vulcanizates with elastic and strength properties that meet the requirements for these industrial rubbers (Table 4). For example, the tensile strength of elastomer compositions with carbon black is 8.1 MPa for rubber profiles and 12.0 MPa for sealing rings, whereas with SM in the specified ratios, these values range from 7.3 to 7.6 MPa and 10.9 to 11.3 MPa, respectively.

A comparative analysis of studies on the sealing and sealing capabilities of rubbers based on SKEPT-50 + SBR-30 ARKM-15 and NBR-28AN, respectively, revealed that when carbon black grade N550 was replaced with shungite mineral, there was a 19.7% reduction in RCS (increased sealing capability) in compositions based on general-purpose rubber combinations and a 16.3% reduction in special-purpose rubber compositions. In the case of full replacement of industrial chalk with SM, the RCS indicator did not change. The identified nature of the RCS improvement in rubbers with SM was attributed to the increased elasticity of the macromolecular segments between the network nodes, promoting a return to the original conformation before compression, i.e., reducing RCS.

A comparative analysis of the strength properties of the studied vulcanizates after thermal aging revealed that under the combined influence of elevated temperatures and oxygen in the presence of SM, structuring processes dominated within the elastomer matrix, leading to improved strength properties of the rubbers. Notably, the elongation at break of the rubbers based on the special-purpose rubber BNR-28AN increased 1.4 times after exposure to high temperatures compared to the industrial composition.

Changes in the properties of rubbers during thermal aging are primarily associated with the spatial structure formed during the vulcanization of the rubber compound.

The results of determining the elastic and strength properties of rubbers based on SKEPT-50 + SBR-30 ARKM-15 (profiles) and SKI-3 + SBR-30 ARKM-15 (mudguards and mats), containing SM as a full replacement for industrial chalk, are presented in Table 11.

It was established that the complete replacement of chalk with SM helps to maintain the strength and elastic properties of rubbers intended for the production of rubber profiles and mats under the influence of elevated temperatures and atmospheric oxygen. At the same time, the full replacement of chalk with the studied mineral in compositions for mudguards leads to an 18.5% increase in tensile strength before thermal aging and a 9.4% increase after exposure to high temperatures.

The observed changes in rubber properties under elevated temperatures and oxygen exposure are attributed to the differing vulcanizate structures formed through random crosslinking of macromolecules due to polymer radical interactions, resulting in branched and crosslinked structures [50].

#### 3.2.2. Investigation of Shore A Hardness of Elastomer Compositions

**Figure 5:**
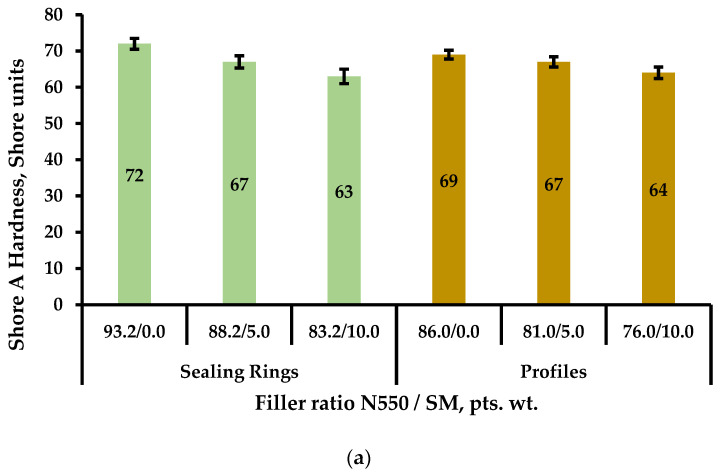

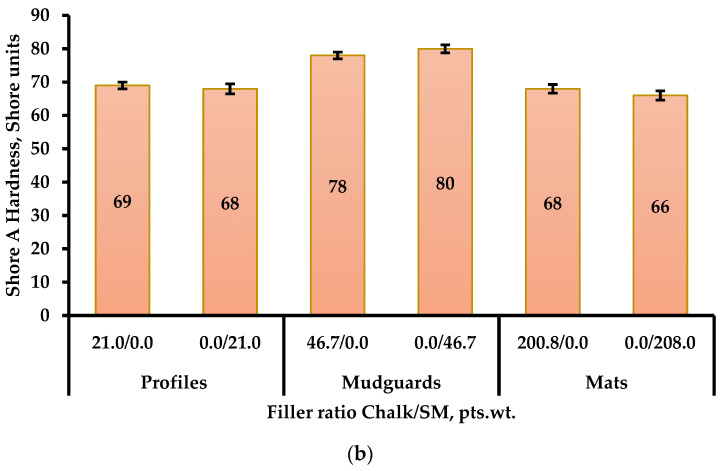
Dependencies of Shore A hardness changes in rubber compounds for various types of RTPs containing (**a**) SM as a partial replacement for N550; (**b**) SM as a full replacement for chalk.

Shore A hardness is a critical parameter that plays an important role in selecting materials for various seals, particularly in hydraulic systems. Figure 5 shows the dependencies of the hardness of the studied rubbers on the type of filler and the degree of filling.

It was determined that replacing the semi-reinforcing carbon black grade N550 with SM at dosages of 5.0 and 10.0 pts. wt. reduced the hardness of rubbers for sealing rings by 5 and 9 Shore A units, respectively (Figure 5a). In contrast, a different trend was observed with the complete replacement of chalk with SM (Figure 5b). Specifically, the full replacement of industrial chalk with the studied mineral in rubbers intended for the production of profiles, mudguards, and mats had virtually no effect on the hardness of the vulcanizates (the change was 3 Shore A units, which falls within the measurement error range).

#### 3.2.3. Determination of Relative Residual Compressive Deformation (RRCD)

One of the key parameters determining the sealing capability of RTPs is elastic recovery after a specified deformation or relative residual compressive deformation (RRCD). This parameter characterizes the ability of a rubber product to retain its elastic properties after being held in a compressed state under specific conditions. RRCD is defined as the residual deformation that occurs below the yield limit of the material. The test for compression measures the ability of rubber to return to its original thickness after prolonged compressive stress at a specified temperature and deformation. Over time, as rubber compresses, it loses its ability to return to its original thickness. This loss of elasticity (“memory”) can significantly reduce the potential service life of such RTPs [51], as they must provide high sealing and compression capabilities.

**Figure 6:**
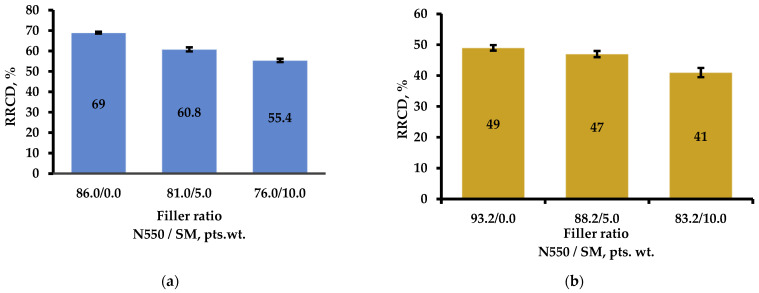
Changes in RRCD of elastomer compositions of various purposes containing SM: (**a**) partial replacement of N550 in formulations for profiles; (**b**) full replacement of chalk in formulations for sealing products.

Figure 6 presents the results of determining the RRCD of the elastomer compositions based on rubbers with different purposes containing SM as a partial replacement for carbon black and a full replacement for chalk.

A comparative analysis of the sealing and gasketing capabilities of rubbers based on SKEPT-50 + SK(M)S-30 ARKM-15 and BNKS-28AN, respectively, revealed that the partial replacement of N550 carbon black with SM, even at relatively low concentrations (5.0 and 10.0 pts. wt.), was accompanied by a noticeable decrease in the compression set indicator (CSR). Moreover, with an increased SM content in the RTP samples, the CSR value continued to decrease. Consequently, the partial replacement of N550 carbon black with SM micropowder contributed to improved elasticity and sealing ability of the RTPs. Indeed, as shown in Figure 6, the CSR indicator decreased by 19.7% in formulations based on a combination of general-purpose rubbers and by 16.3% in formulations based on special-purpose rubbers.

The observed improvement in the RRCD of the studied rubbers partially filled with SM micropowders is likely due to the unique composition and structure of SM, which is inherently a carbon–silicon material with bipolar properties. These characteristics provide high thermodynamic compatibility of this material with rubbers of any nature. The presence of microparticles of such a specific reinforcing filler as SM in the structure of RTPs likely enhances the elasticity of the macromolecular segments between the crosslinking nodes of the polymer network. This contributes to the rubber’s ability to return to its original conformation after compression, thereby reducing the RRCD.

#### 3.2.4. Study of Rubbers with Shungite Mineral for Resistance to Liquid Aggressive Media

Rubber technical products are used in various industries, and one of the specific requirements for them is low swelling when exposed to aggressive media. Experimental measurements are the best way to determine the sorption of swelling agents in polymers. Rubbers possess two properties that distinguish them from most other typical solids: they can absorb large amounts of solvent without dissolving and can undergo significant deformations at relatively low stresses [52]. The properties of elastomers change when exposed to various chemicals. One of the most common effects is physical swelling due to the presence of a cross-linked network structure. Depending on the base elastomer and aggressive medium, the degree of swelling varies from negligible to significant, and can reach several hundred percent. Aggressive liquids interact with the chemical ingredients in elastomers, thus affecting their mechanical or technological properties. The swelling of elastomers can be used to simply increase the volume or functional effects such as self-healing. Vulcanizates exhibit dynamic changes in volume and thickness when exposed to swelling media like water, oils, and acids [53].

**Figure 7:**
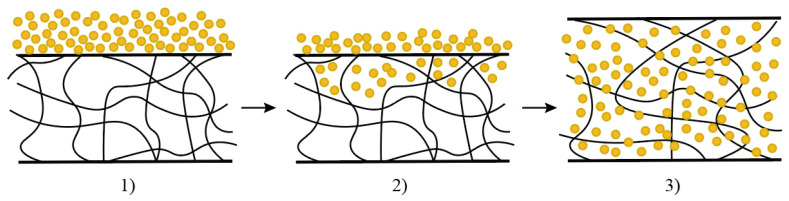
Absorption of aggressive liquid media by elastomer through the diffusion process.

Oil- and gasoline-resistant rubbers swell by absorbing hydrocarbons through a diffusion process. The swelling mechanism can be illustrated as a combination of three separate processes (Figure 7):

1. Absorption of the solvent on the polymer surface;
2. Penetration of the solvent into the polymer, initially by filling pores and free volume, followed by diffusion of solvent molecules into the polymer;
3. The polymer structure expands as the solvent absorbed into the pores penetrates the network of polymer chains, causing them to swell [6].

Elastomer compositions based on BNR-28AN are used as sealing products, making resistance to liquid aggressive media an important characteristics for such types of products. Table 12 presents the results of determining the change in mass of vulcanizates based on special-purpose rubber containing SM as a partial replacement for N550 after exposure to an “isooctane” (70:30) mixture.

The results of determining the resistance of elastomer compositions based on special-purpose rubber BNR-28AN revealed that the use of SM in dosages of 5 and 10 pts. wt. for partial replacement of semi-reinforcing carbon black grade N550 led to an increase in the resistance of vulcanizates to aggressive media by 8.9–15.6%. The observed change in the mass of rubbers with partial replacement of carbon black after swelling in the “isooctane” mixture was likely because the studied mineral does not chemically interact with the aggressive medium and was wetted similarly or less effectively than rubber. Consequently, its introduction reduced the rubber’s volumetric content and lengthened the aggressive medium’s diffusion path [50].

## 4. Conclusions

The possibility of partially replacing carbon black with SM micropowder as fillers in industrial formulations of elastomer compositions intended for the production of RTPs for various purposes was studied.

In this study, SM was used as high-dispersion micropowder, which was produced from ore mined in East Kazakhstan, Republic of Kazakhstan. This material is characterized by bipolar properties due to its high carbon and silicon content. Due to the unique composition and structure of SM micropowders, their use as fillers in industrial elastomer formulations significantly improves the physical and mechanical properties of RTPs manufactured using such compositions.

It was demonstrated that the partial replacement of semi-reinforcing carbon black grade N550 with SM reduced the Mooney viscosity of rubber compounds by up to 26.8%, decreased the vulcanization optimum time by up to 23.7%, and lowered the compression set indicator (CSR) for the RTPs by up to 19.7% and 16.3%, respectively. This indicates an improvement in the sealing and gasketing properties of the RTPs. When SM micropowders were used to replace industrial chalk completely, it was found that this substitution had little to no impact on the kinetic parameters of rubber compound processing. However, it increased the strength of the RTPs by up to 18.5% and enhanced their resistance to liquid aggressive environments.

Thus, this study confirmed the feasibility of using SM from East Kazakhstan in rubber product formulations as a partial replacement for N550 carbon black at dosages of up to 10.0 pts. wt. and as a complete replacement for chalk. This reduces the final products’ production cost and improves their physical and mechanical properties.

This research has future potential and could focus on the following key areas: exploring the development of hybrid fillers combining SM with other reinforcing agents to further enhance the desired properties; evaluating the scalability of using shungite-based fillers in industrial settings, focusing on production efficiency, cost-effectiveness, and consistency in quality; performing real-world testing of RTPs containing shungite-based fillers in specific industries, such as the automotive, construction, or oil and gas industries, to validate the laboratory findings; and investigating the environmental impact of producing and using shungite-based fillers, with the aim of developing sustainable production processes.

## Figures and Tables

**Table 1:** Formulations of industrial rubber compounds for RTP production.

Ingredient Name	The Content of Ingredients, pts. wt. per 100 pts. wt. of Rubber			
	Profiles	Mudguards	Mats	Sealing Rings
SKEPT-50	20.0	–	–	–
SBR-30 ARKM-15	80.0	23.0	42.0	–
IR (SKI-3)	–	55.0	58.0	–
BNR-28AN	–	–	–	93.0
Zinc white	6.0	1.8	2.3	6.8
Acetoanil N	2.1	1.5	–	0.9
Diafen IPPD	2.1	1.5	–	0.9
Stearic acid	2.1	–	2.3	0.5
Carbon black N550	86.0	90.0	95.4	93.2
Chalk	21.0	46.7	200.8	–
Calcium naphthenate M1	7.1	–	–	–
Santogard PVI	1.0	0.3	–	–
Oil I-20A	25.5	11.0	–	–
Protective wax	3.4	2.1	2.3	2.3
PEG-4000	2.9	–	–	–
Sulfur	2.6	0.6	1.5	–
Diphenylguanidine (DFG)	0.8	–	–	–
Sulfenamide C	2.1	–	–	0.5
Regenerate	–	55.0	143.5	–
Thiuram D	–	0.6	0.8	0.5
Altax	–	1.2	0.8	–
Petroleum bitumen	–	9.8	16.0	–
Benzoic acid	–	0.3	0.8	–
Softener	–	–	61.8	–
Rubber crumb SP	–	–	137.4	–
DBP (dibutyl phthalate)	–	–	–	0.9
DTDM (dithiodimorpholine)	–	–	–	0.5

**Table 2:** Properties of carbon black according to ASTM D1765-03 [40].

Classification (ASTM)	Iodine Adsorption Number, g/kg	DBP Absorption, cm³/100 g	pH of Aqueous Suspension	Surface Area for Multi Point Nitrogen Adsorption, m²/g	External Surface Area for Nitrogen Adsorption, m²/g	Bulk Density, kg/m³
N550	43 ± 4	101 ± 4	6.0–8.0	41	39	360

**Table 3:** Properties of industrial chalk.

Characteristic	Numerical Value, Standardized According to Technical Specifications (TS)
Total mass fraction of calcium carbonate and magnesium carbonate (calculated as calcium carbonate), minimum	98.5%
Mass fraction of substances that are insoluble in hydrochloric acid, maximum	1.3%
Mass fraction of iron and aluminum oxides, maximum	0.4%
Mass fraction of moisture, maximum	0.2%
Reflectance coefficient, minimum	86.0%

**Table 4:** Technical specifications for RTPs.

Characteristic	RTP Type			
	Profile	Mudguards	Mats	Sealing Rings
Tensile strength, MPa, minimum	5.9	9.8	4.0	10.8
Elongation at break, %, minimum	180	250	110	150
Hardness, Shore A units	60–80	70–75	60–75	75–85
Compression set at 20–30% compression, %, maximum	70.0	–	–	60.0
Change in mass after exposure to a 70:30 by volume mixture of isooctane and toluene for 24 h at (23 ± 3) °C, %, maximum	–	–	–	25.0

**Table 5:** Physical–chemical characteristics of SM.

Characteristics	Value
Appearance	Highly dispersed gray powder
Bulk density, kg/m³	500–600
Grinding fraction	15.0 ÷ 35.0 µm
Moisture content, %	0.5–1.5
Loss on ignition, %	8–14
pH of aqueous extract	7–9
Dibutyl phthalate absorption, cm³/100 g	28–35
Mass fraction of residue after sieving using sieve No. 0045	Less than 0.08
Specific external surface area by nitrogen adsorption, m²/g	23–25

**Table 6:** Elemental composition of the surface of SM.

Elements	Content, % *w/w*
C	17.16
O	50.28
Si	16.52
Al	9.68
K	1.98
Fe	2.11
Na	0.88
Mg	0.57
Ca	0.43
Ti	0.39

**Table 7:** Filler ratios in industrial elastomer compositions.

Product Name			
Profiles	Mudguards	Mats	Sealing Rings
Partial Replacement of Carbon Black N550 with Shungite Mineral			
Ratio of N550/SM, pts. wt. per 100 pts. wt. of rubber			
86.0/0.0 (industrial mix)	–	–	93.2/0.0 (industrial mix)
81.0/5.0 (experimental mixture)	–	–	88.0/5.0 (experimental mixture)
76.0/10.0 (experimental mixture)	–	–	83.2/10.0 (experimental mixture)
Complete Replacement of Chalk with SM			
Ratio of Chalk/SM, pts. wt. per 100 pts. wt. of rubber			
21.0/0.0 (industrial mix)	46.7/0.0 (industrial mix)	200.8/0.0 (industrial mix)	–
0.0/21.0 (experimental mixture)	0.0/46.7 (experimental mixture)	0.0/200.8 (experimental mixture)	–

**Table 8:** Kinetic characteristics of rubber compounds with partial replacement of N550 with SM.

Type of Product	Filler Ratio N550/SM, pts. wt.	Kinetic Parameters		
		*t_s_*_2_, min	*t*_90_, min	Δ*M*, dN∙m
Rubber profiles	86.0/0.0 (industrial mixture)	1.46 ± 0.07	3.29 ± 0.16	22.4 ± 1.1
	81.0/5.0 (experimental mixture)	1.15 ± 0.06	2.93 ± 0.15	18.5 ± 0.9
	76.0/10.0 (experimental mixture)	1.02 ± 0.05	2.51 ± 0.13	16.4 ± 0.8
Sealing rings	93.2/0.0 (industrial mixture)	1.27 ± 0.06	2.51 ± 0.13	35.6 ± 1.8
	88.2/5.0 (experimental mixture)	1.28 ± 0.06	2.51 ± 0.13	33.4 ± 1.7
	83.2/10.0 (experimental mixture)	1.3 ± 0.6	2.49 ± 0.12	31 ± 2

Notes: *t_s_*_2_—time required for the minimum torque to increase by 2 units, min; *t*_90_—time to reach the optimal vulcanization level, min; Δ*M*—difference between maximum and minimum torque, dN·m.

**Table 9:** Kinetic characteristics of rubber compounds with full replacement of chalk with SM.

Type of Product	Filler Ratio N550/SM, pts. wt.	Kinetic Parameters		
		*t_s_*_2_, min	*t*_90_, min	Δ*M*, dN∙m
Rubber profiles	86.0/0.0 (industrial mixture)	1.46 ± 0.07	3.29 ± 0.16	22.4 ± 1.1
	0.0/86.0 (experimental mixture)	1.44 ± 0.07	3.22 ± 1.16	22.3 ± 1.1
Mudguards	46.7/0.0 (industrial mixture)	0.77 ± 0.04	1.32 ± 0.07	21.2 ± 1.1
	0.0/46.7 (experimental mixture)	0.72 ± 0.04	1.24 ± 0.06	20.8 ± 1.0
Mats	200.8/0.0 (industrial mixture)	0.63 ± 0.03	0.93 ± 0.05	13.4 ± 0.7
	0.0/200.8 (experimental mixture)	0.57 ± 0.03	0.87 ± 0.04	13.5 ± 0.7

Notes: *t_s_*_2_—time required for the minimum torque to increase by 2 units, min; *t*_90_—time to reach the optimal vulcanization level, min; Δ*M*—difference between maximum and minimum torque, dN·m.

**Table 10:** Elastic and strength properties (before and after thermal aging) of rubbers based on SKEPT-50 + SBR-30 ARKM-15 and BNR-28AN with SM.

Type of Product	Filler Ratio N550/SM, pts. wt.	*f_p_*, MPa		*ε_p_*, %	
		Before Aging	After Aging	Before Aging	After Aging
Rubber profiles	86.0/0.0 (industrial mixture)	8.1 ± 0.8	8.4 ± 0.8	290 ± 29	190 ± 19
	81.0/5.0 (experimental mixture)	7.6 ± 0.8	7.7 ± 0.8	340 ± 34	230 ± 23
	76.0/10.0 (experimental mixture)	7.3 ± 0.7	7.5 ± 0.7	380 ± 38	250 ± 25
Sealing rings	93.2/0.0 (industrial mixture)	12 ± 1	12.8 ± 1.3	160 ± 16	190 ± 19
	88.2/5.0 (experimental mixture)	11.3 ± 1.1	13.7 ± 1.4	140 ± 14	230 ± 23
	83.2/10.0 (experimental mixture)	10.9 ± 1.1	14.1 ± 1.4	130 ± 13	260 ± 26

**Table 11:** Elastic and strength properties (before and after thermal aging) of rubbers based on SKEPT-50 + SBR-30 ARKM-15 and SKI-3 + SBR-30 ARKM-15 with SM.

Type of Product	Filler Ratio Chalk/SM, pts. wt.	*f_p_*, MPa		*ε_p_*, %	
		Before Aging	After Aging	Before Aging	After Aging
Rubber profiles	86.0/0.0 (industrial mixture)	8.1 ± 0.8	8.4 ± 0.8	290 ± 29	190 ± 19
	0.0/86.0 (experimental mixture)	8 ± 1	7.9 ± 0.8	300 ± 30	210 ± 21
Mudguards	46.7/0.0 (industrial mixture)	10.3 ± 1.0	10.6 ± 1.1	340 ± 34	150 ± 15
	0.0/46.7 (experimental mixture)	12.2 ± 1.2	11.6 ± 1.2	330 ± 33	190 ± 19
Mats	200.8/0.0 (industrial mixture)	4.5 ± 0.4	3.7 ± 0.4	240 ± 24	150 ± 15
	0.0/200.8 (experimental mixture)	8.1 ± 0.8	8.4 ± 0.8	290 ± 29	190 ± 19

**Table 12:** Change in mass of rubbers based on NBR-28AN after exposure to aggressive liquid.

Product Type	Ratio of Fillers N550/SM. pts. wt.	Change in Mass of Vulcanizate, %
Sealing rings	93.2/0.0 (industrial mix)	18.0
	88.2/5.0 (experimental mixture)	16.4
	83.2/10.0 (experimental mixture)	15.2

## Data Availability

The data presented in this study are available on request from the corresponding author due to the privacy restrictions.
